# Assessment of the Suitability of Flour Obtained from Mountain Rye Grain Milling and the Method of Dough Fermentation for the Production of Rye Bread

**DOI:** 10.3390/foods13193035

**Published:** 2024-09-24

**Authors:** Joanna Kaszuba, Magdalena Czyż, Tomasz Cebulak, Karolina Pycia

**Affiliations:** Department of Food Technology and Human Nutrition, Institute of Food Technology and Nutrition, College of Natural Science, University of Rzeszow, Zelwerowicza Street 4, 35-601 Rzeszow, Poland; jkaszuba@ur.edu.pl (J.K.); tcebulak@ur.edu.pl (T.C.)

**Keywords:** rye, sourdough, baking value, amylographic analysis, bread quality assessment

## Abstract

Currently, there is an increase in consumer interest in food produced from raw materials from organic farming, which has an impact on the greater attention paid to the possibility of increasing the cultivation of old cereal species. One of the cereals that is suitable for these trends is mountain rye, which is a premise for undertaking research on the usefulness of this cereal grain in food production. Therefore, the aim of the study was to compare the baking value of flour with different milling yields obtained from milling mountain rye grain. The research material consisted of rye grain (*Secale montanum* Guss.), which was milled, and 6 different rye flours were obtained. The flour was tested for selected quality parameters such as moisture, crude protein content, total ash content, and acidity. Doughs were prepared and fermented using a single-phase method carried out in two different variants, with or without the addition of lactic acid. The obtained rye breads were quality assessed and subjected to organoleptic and consumer evaluations. The use of the fermentation method with dough acidification with lactic acid allowed us to obtain breads with a better specific volume and acidity compared to those obtained from dough without acidification. Breads baked from dough prepared using the method of non-acidification with lactic acid had better porosity of the crumb. In the quality classification, breads made from low-extract flour turned out to be the best, and breads baked from dough made using the non-lactic acid acidification method were more generally accepted by panelists. As confirmed by research, mountain rye grain is a raw material for the production of flour with good baking value, which depends on the preparation of the grain and milling method. The quality of rye bread made from mountain rye flour depends on the flour yield, the baking value of the flour, and the method used for dough fermentation for bread baking

## 1. Introduction

Cereal grains are the most valuable source of energy in human nutrition because they are a source of digestible complex carbohydrates. They also provide the human body with proteins, unsaturated fatty acids, B vitamins, and minerals such as magnesium, manganese, phosphorus, iron, zinc, and copper. Rye grain is also a source of fiber, including valuable functional ingredients such as pentosans and β-glucan [1,2]. In addition, cereal grains and their products enrich the diet with antioxidants. Rye grain is also a rich source of various phytochemicals, such as phenolic acids, lignans, and alkylresorcinols, but also flavonoids, anthocyanins, phenolamides, and benzoxazinoids [3,4]. Rye (*Secale cereale* L.) was not always considered a grain. Initially, it was referred to as a noxious weed, but over time, its fertility and resistance to diseases and unfavorable weather conditions were noticed. Due to its well-developed root system, rye has low water requirements, which helps it survive in water-poor soil and even in drought conditions. It is tolerant to acidic soil, and losses in the physicochemical properties of the soil do not affect the proper development of plants and grains. Among other cereals, it stands out for its resistance to low temperatures (even below −25 °C) [5,6,7]. The area of rye cultivation in the world is about 11 million ha, which, in the total area of all cereals sown, is only 1.5%. Europe dominates the production of rye grain in the world, as only 5% of the world production of this grain is in non-European countries. According to data from the UN Food and Agriculture Organization (FAO), in 2022, the significant producers of this grain were Germany, Poland, Russia, Belarus and Denmark, followed by Canada. Currently, Polish rye harvests account for 20% of the entire world production, which makes Poland a very big competitor to its western neighbors. The total area of cereal cultivation in Poland in 2020 amounted to about 7.9 million ha, while rye was about 0.9 million ha. The rye harvest in 2020 amounted to 3.1 million tons, which was the best result in the last 10 years [8]. The use of rye grain depends on its features and properties. An important direction of using rye grain is feed production for farm animals, if only because it beats other cereals in terms of the cost of cultivation. The grain of this species has a favorable amino acid balance, despite its low protein content. At the same time, it has many antinutritional substances that can negatively affect the development of young pigs or cattle [9,10].

The main direction of rye grain use is the milling and baking sector, i.e., the consumer direction. In the consumer sector, in addition to bread and flour, flakes are produced, as well as extruded products, e.g., crisp bread or instant pasta. Prepared rye grain can be used interchangeably with rice, groats and, to some extent, wheat flour [11,12]. Another equally popular direction of using rye grain is the production of ethanol and kvass [11,13]. An important direction is the processing of grain into malt, which is used in brewing, among other applications [14].

The most important components that shape the structure of dough and the quality of bread made from rye flour are starch, pentosans and protein. Rye bread, in comparison to wheat bread, is poorer in carbohydrates. The most important carbohydrate is starch, which has the properties of binding water at the stage of dough formation and preserving it during baking. Due to the ability of starch to gelatinize, i.e., to create a sticky mass, the finished product has greater absorption by the digestive system [15,16]. It has been shown that the evaluation of the baking quality of today’s commercially produced rye flour should include a swelling curve test, together with informative parameters regarding the starch–amylolytic system, such as the initial and final starch gelatinization temperature. Such an evaluation provides a better way to determine the baking quality of commercial rye flour and its suitability for the production of good quality rye bread [17]. Sourdough rye bread, in comparison to wheat bread made from sourdough, is characterized by a lower glycemic index. The consumption of this type of bread lowers blood glucose levels, which is why diabetics should reach for it more often. In addition, the lactic acid bacteria present in sourdough bread make sourdough bread also have antioxidant properties. It is worth noting that eating rye bread is not recommended for every consumer. People on a easily digestible diet or suffering from digestive tract diseases such as stomach ulcers, duodenal ulcers, or diverticulosis, as well as people who have undergone surgeries, procedures, and infections, should not eat this type of bread, because it is difficult to digest and may lead to digestive problems [18,19].

Mountain rye (*Secale montanum* Guss.) is one of the oldest but forgotten cereal species. It has much in common with St. John’s Eve and traditional slash-and-burn farming. It is currently referred to as ancient and unrefined rye. It is a cereal that is resistant to diseases, climatic and soil conditions, which is why it can be grown on organic farms. In the past, the grain was mainly used as animal feed and bread and dumplings were baked from common rye flour only on a few farms. The great similarity of mountain rye to common rye makes them easy to crossbreed. Until the 19th century, mountain rye was widely cultivated throughout Central Europe. However, due to the low yields (1.5–3.0 tons/ha), dark color of grain, and easy falling out of the ear, this species was replaced by modern common rye [20,21]. Studies by other authors have shown that mountain rye flour has a comparable baking value to common rye flour, but mountain rye grain contains more protein, free phenolic compounds, and ash, and has a lower starch content and a similar antioxidant potential compared to common rye grain. The results of the amylographic test showed that rye flour from mountain rye grain was characterized by a high baking quality [22,23]. Bread from mountain rye grain flour was successfully used to produce kvass [24].

Consumer interest in food produced from raw materials from organic farming encourages greater attention to the possibility of increasing the cultivation of old cereal species and introducing them to the food market. In connection with the above, the aim of this study was to assess and compare the baking value of flour with different extracts obtained from mountain rye grain using various methods, including indirect methods such as testing the physicochemical properties of flour, including starch, and the direct bread baking method, and then assess the baking process parameters, bread quality parameters, and its sensory properties in order to develop technological recommendations for rye grain milling, dough preparation and bread baking.

## 2. Materials and Methods

### 2.1. Raw Material

The research material was mountain rye grain (*Secale montanum* Guss.), which, in Polish, is krzyca, from the 2019 harvest from organic farming (51°20′14″ N 18°56′29″ E) in Dąbrowa Rusiecka (Bełchatów County, Łódzkie Voivodeship, Poland), and 6 different types of mountain rye flour from the milling of this grain made in the Cereal Grain Processing Technological Line of the University of Rzeszów (Poland). The grain and flours intended for the research were stored in a warehouse (temperature of 12 °C).

### 2.2. Methods

#### 2.2.1. Preparing Flour for Testing

Before milling, the grain was cleaned in the SLN3 sorter (Pfeuffer, Kitzingen, Germany) and 24 kg of rye grain was intended for milling. The grain was then divided into two portions: (1) unconditioned grain (S) with a moisture content of 12.8% and (2) grain intended for conditioning to a moisture content of 14.0%. The conditioned grain (K) matured in glass jars for 12 h. Unconditioned rye grain was divided into three portions and subjected to grinding in three variants, and as a result, three types of flour were obtained: wholegrain flour (S MC) obtained by grinding grain in a LabMill laboratory roller mill (Perten, Huddinge, Sweden), extract flour (S MBO) obtained after milling rye grain in a Quadrumat Junior laboratory hammer mill (Brabender, Duisburg, Germany), flour with bran (S MO) obtained by milling grain in a Quadrumat Junior laboratory roller mill (Brabender, Duisburg, Germany), and the obtained flour was combined with bran, which was additionally ground in a LabMill laboratory hammer mill (Perten, Huddinge, Sweden).

**Figure 1:**
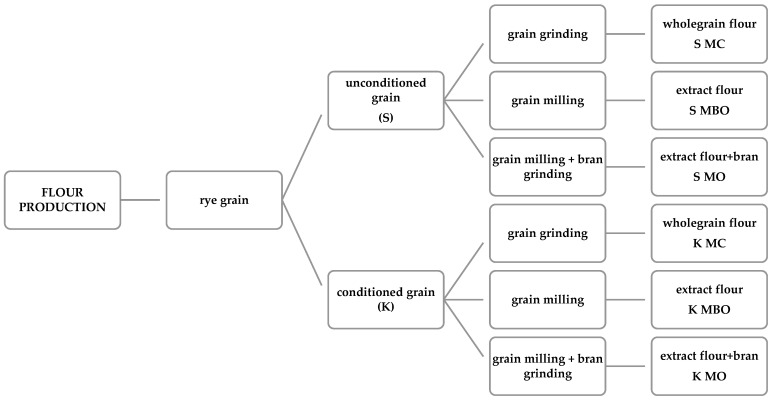
The mountain rye flour production scheme.

The second batch of rye grain, which had been conditioned, was divided into three portions and sent for grinding in three variants. As a result, three different types of flour were obtained: wholemeal flour (K MC) obtained by grinding the grain in a LabMill laboratory hammer mill (Perten, Huddinge, Sweden), extract flour (K MBO) obtained after milling the grain in a Quadrumat Junior laboratory roller mill (Brabender, Duisburg, Germany), and flour with bran (K MO) obtained by milling the grain in a Quadrumat Junior laboratory roller mill (Brabender, Duisburg, Germany), and then the bran was ground again in a LabMill hammer mill (Perten, Huddinge, Sweden) and combined with the flour. The flour production scheme is presented in Figure 1.

All flours were placed in paper bags and stored in a warehouse (temperature of 12 °C) until the analyses.

#### 2.2.2. Research on Selected Quality Parameters of Mountain Rye Flour

Rye grain intended for the moisture determination was crushed in a Cemotec mill (FOSS, Hilleroed, Denmark). The moisture content (%) of the grain was determined using a MAC50 moisture analyzer (Radwag, Radom, Poland) in triplicate.

A laboratory flat sieve (Sadkiewicz Instruments, Bydgoszcz, Poland) and a set of sieves with the following equivalent mesh diameters were used to conduct the flour sieve analysis: 132 μm, 150 μm, 180 μm, 265 μm, and 315 μm. The flour fractions were collected as a fraction from each sieve and a sieve sifting from a 132 μm sieve and weighed to the nearest 0.1 g.

The moisture content of the flour was determined according to AACC method 44-15.02 [25] using a Binder convection dryer (Tuttlingen, Germany) at 130 °C for 1 h. The falling number determines the activity of the alpha-amylase enzyme, which occurs in small amounts in ripe grain that was harvested under dry conditions. To determine the falling number of flours, i.e., the falling time of the stirrer in the suspension made of flour and water, which depends on the alpha amylase activity in the tested flour and thus on the liquefaction rate of the produced starch paste, a Falling Number apparatus from Perten (Huddinge, Sweden) was used and the determination was performed according to ISO 3093:2009 [26]. The nitrogen content was determined by the Kjeldahl method (Kjeltec TM 8400, Hilleroed, Denmark), the crude protein content was calculated according to AACC Method No. 46-10 [25] using the Nx6.25 converter, and the total ash content was calculated by incinerating the flour sample in a P330 muffle furnace (Nabertherm, Lilienthal, Germany) according to AACC Method No. 46-11.02 [25]. A viscograph-E (Brabender, Duisburg, Germany) and ICC standard method No. 169 guidelines [27] were used to assess the amylographic properties of rye flour. For this purpose, a 15% suspension of flour in water was prepared and heated at a rate of 1.5 °C/min in the range of 30–95 °C. The following parameters were recorded: beginning gelatinization temperature (°C), maximum viscosity (BU, Brabender Unit), and end gelatinization temperature (°C). The potential acidity of flour was determined by titration [28,29] and given in acidity degrees (one degree means the number of cm^3^ of a 1 M NaOH solution used to neutralize the acids contained in 100 g of rye flour).

#### 2.2.3. Dough Fermentation and Baking of Mountain Rye Bread

**Figure 2:**
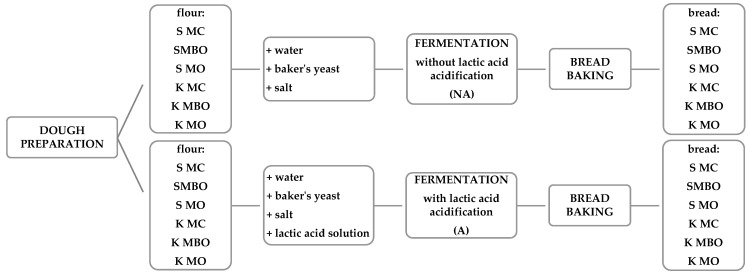
Scheme of the bread making process.

A laboratory test baking of rye flour was carried out using the direct (single-phase) method with baker’s yeast in two variants: without acidification with lactic acid (NA) and with acidification (A) [28]. The dough was made from a flour mass corresponding to the dry matter content in 350 g of flour with a moisture content of 15%, 8 cm^3^ of a 1 M solution of lactic acid (Biomus Sp. z o.o., Lublin, Poland), tap water to the volume required to obtain a dough yield of 165%, yeast in the amount of 3%, and salt in the amount of 1.5% of the flour mass. After mixing, the dough was fermented for 1 h at 35 °C in a fermentation chamber (humidity 60%) (Sveba Dahlen, Fristad, Sweden). After the fermentation was complete, the dough was weighed, divided into 350 g pieces, shaped into a ball, then placed in greased metal baking tins and subjected to final fermentation. The risen dough pieces were placed in the baking chamber of a Classic electric oven (Sveba Dahlen, Fristad, Sweden). Baking was carried out at 240 °C for 40 min. The breads were removed from the oven and then from the tins. The hot loaves were weighed and left to cool. The bread baking process diagram is shown in Figure 2.

#### 2.2.4. Mountain Rye Bread Quality Assessment

The specific volume of the bread was calculated from the measurement of loaf weight and volume [30] using a Sa-Wa bread volumeter (Sadkiewicz Instruments, Bydgoszcz, Poland). The crumb moisture content was determined according to AACC Method No. 44-15.02 [25]. The crumb porosity (%) of the rye breads tested was determined by determining the difference in volume between the intact crumb and the crumb deprived of pores by kneading [28]. Crumb acidity (total titratable acidity—TTA) was determined according to AACC Method No. 02-31.01 [25].

#### 2.2.5. Sensory and Consumer Evaluations of Mountain Rye Bread

Before the tests, 10 trained panelists received information about the study’s purpose and gave their consent in accordance with the university’s ethics committee. The socio-demographic profile of the panelists was not recorded.

The sensory analysis of the bread was performed using the point method according to the PN-A-74108 standard [31] and as described by Krochmal-Marczak et al. [32]. The assessment took into account parameters such as the external appearance, color and thickness of the crust, elasticity and porosity of the crumb, and taste and smell of the product. Physicochemical properties were also taken into account, i.e., acidity, moisture, and volume, and then presented in a score range in accordance with PN-A-74108 [31]. The quality classification was made based on the criteria provided in the mentioned standard [31]. The study was conducted three times using an anonymous form.

Consequently, the overall acceptability of tested bread samples was determined. In order to determine the degree of consumer desirability of the rye breads studied, a 9-point hedonic scale was used (1—dislike extremely, 2—dislike very much, 3—dislike, 4—dislike slightly, 5—neither like nor dislike, 6—like slightly, 7—like, 8—like very much, and 9—like extremely). The study was conducted three times using an anonymous form.

#### 2.2.6. Statistical Analysis of the Results

The analyses and baking of breads were performed in triplicate. The results of the conducted studies were statistically processed using Statistica ver. 13.3 software (TIBCO Software Inc., Palo Alto, CA, USA) and Microsoft Excel 2019 spreadsheets (Microsoft, Redmond, WA, USA). The analysis included calculating the arithmetic mean values ± standard deviations. To compare the differences in mean values, a one-way analysis of variance (ANOVA) was performed using Duncan’s test at a significance level of *p* = 0.05. The graphical interpretation of the results was performed using scaled heat maps created in RStudio ver. 2023.09.0 + 463 software (RStudio Team, Posit, PBC).

## 3. Results and Discussion

The baking value is a set of flour features that describe the properties of flour and dough and allow predictions of the quality of bread made from it. An appropriate evaluation of the functional properties of the main components of rye flour and the measurement of the activity of enzymes responsible for their degradation allows the characterization of the baking value of rye flour [33].

### 3.1. Quality Parameters of the Tested Mountain Rye Flours

Table 1 presents the results of the quality parameters of the mountain rye flours tested.

Based on the statistical analysis of the results, it was found that the tested mountain rye flours differed slightly in terms of the moisture content. The moisture content ranged from 12.05 to 13.50%. Flour obtained by grinding mountain grain in a laboratory hammer mill (K MC) only differed statistically from flour with bran (S MO) and (K MBO) in terms of the moisture content.

The statistical analysis of the crude protein content in the tested mountain rye flours showed a significant difference between the flours in terms of this parameter. A smaller protein content was recorded in flours without bran, both of which were obtained from unconditioned grain (S MBO) and conditioned grain (K MBO), and no statistically significant difference in the content of the discussed component was shown. However, these flours differed statistically significantly from the other tested mountain rye flours. The results are consistent with the results of previous studies, which confirmed that the chemical composition of flour depends on its extraction because the distribution of components in rye grain is uneven [34]. The tested wholegrain rye flours (MC) and flour with bran (MO), regardless of whether the grain was moistened before milling, showed similar total ash contents. Only extract flours without bran (MBO) differed in terms of the ash content, with conditioned grain flour containing the least amount of the discussed component among all rye flours tested (Table 1). Similar ash contents in rye flour were obtained by Stępniewska [35].

The tested mountain rye flours differed statistically significantly (*p* ≤ 0.05) in terms of acidity, which is related to the content of the grain cover fragment in the flour. Among the tested rye flours from krzyca rye grain, the lowest acidity was noted for flours without bran, both from unconditioned and conditioned grain. On the other hand, the highest acidity was noted for flours obtained from conditioned grain, both wholegrain flour and flour with bran. Based on results from the literature, the acidity of rye flour is related to the content of cover fragments in the flour [33], which was confirmed in the discussed studies of rye flours from mountain rye grain with different total ash contents (Table 1).

The differences noted in the discussed selected rye flour quality parameters were caused by different extracts of the tested flour. Due to the fact that the composition of rye grain is not uniform, the amount of the degree of separation of the fruit-seed coat translates into the chemical composition of the flour and its acidity. Additionally, the greater the share of grain components in the flour, the higher the ash and protein contents that were noted. This is consistent with the results of research by other authors [36,37] who found influences of the rye grain milling method and the yield of the final product on the chemical composition and baking value of rye flour.

### 3.2. Granulation of the Tested Rye Flours

**Figure 3:**
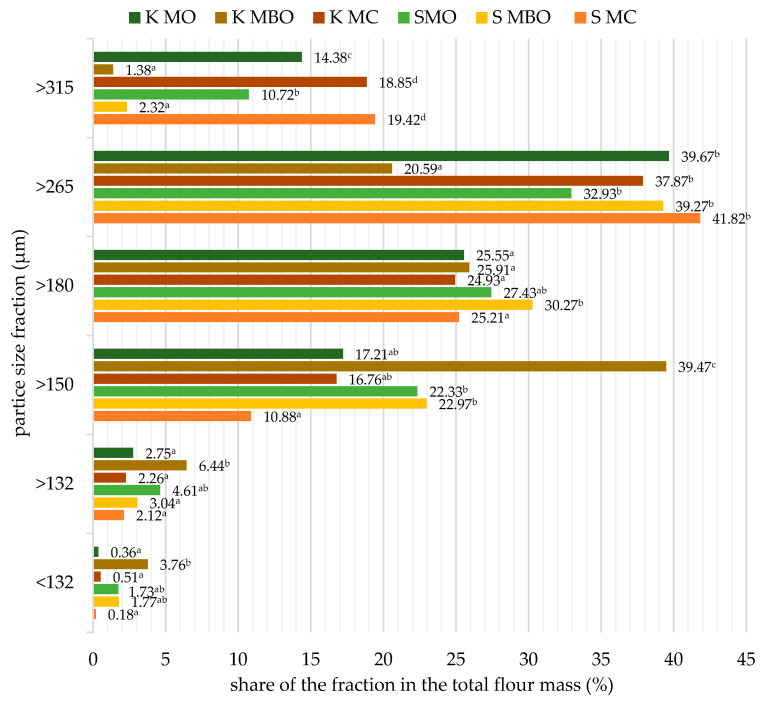
Particle size distribution of the tested mountain rye flours. Mean values in columns marked with different letters differ statistically significantly at a significance level of *p* ≤ 0.05. Abbreviations: S MC—wholegrain flour from grinding unconditioned grain, S MBO—extract flour from milling unconditioned grain, S MO—flour made from a combination of extract flour and bran after milling unconditioned grain, K MC—wholegrain flour from grinding conditioned grain, K MBO—extract flour from milling conditioned grain, K MO—flour made from a combination of extract flour and bran after milling conditioned grain.

Flour granulation is defined as one of the quality parameters. Its value, depending on the size, can significantly affect the fermentation of dough and the quality of rye baking [38]. The granulometric distribution of the tested rye flours obtained from the grinding of rye grain in different variants is presented in Figure 3.

In the tested flours from mountain rye grain (Figure 3), the share of particles in the particle size fraction > 315 μm was similar and, at the same time, the smallest in the bran-free flours (K MBO and S MBO), and they also differed statistically significantly (*p* < 0.05) from the other types of krzyca rye flour tested. The highest share of particles in the mentioned fraction was noted in wholegrain flours (S MC and K MC), which did not differ statistically significantly. The factor that could have caused the discussed share of particles of the tested mountain rye flours in this flour fraction was the different share of grain cover in the flour and the method of grain grinding. The share of particles in the >265 μm size fraction was similar in most of the rye flours tested, and only the extract flour obtained from conditioned grain (K MBO) differed from the others, with a significantly smaller content of particles in the >265 μm size fraction. In the >180 μm particle size fraction, similarly to the one discussed earlier, the share of particles in the tested flours was similar, except for the flour S MBO. A comparison of the results of the share of flour particles in the particle size fraction > 150 μm allowed us to notice that in K MBO flour, the share of particles was significantly largest and almost twice as large as in the other krzyca flours tested. Equally, in K MBO flour, the largest share of particles in the fraction > 132 μm was noted. The other mountain rye flours tested did not differ in the share of particles in this flour particle size fraction. Taking into account the particle size fraction above 150 μm, K MBO flour differed statistically significantly from the other rye flours tested. Grain that is moistened before milling crumbles more easily, and the resulting difference in moisture between the endosperm and the outer layer enables the separation of the fairly elastic outer layer and the endosperm is better crushed. A comparison of the granulometric distribution of the krzyca rye flours tested allowed us to state that the flour without bran from conditioned grain (K MBO) turned out to be significantly different from the others. The obtained results are consistent with the literature data, as it was confirmed that the moisture content of the grain used for grinding is a value that shapes the granulometric distribution of the product. The moisture content of grain intended for milling also influences the flour extract and the degree of starch damage [38].

### 3.3. Falling Number and Amylographic Parameters of Mountain Rye Flour

Table 2 presents the results of the falling number determination and amylographic analysis of the tested mountain rye flours.

In grain characterized by a moisture content above 15% that was formed in unfavorable weather conditions, the alpha-amylase enzyme is activated, which can cause serious damage to starch. The milling process of such grain can lead to the formation of flour with unfavorable physicochemical properties and subsequently to the production of poorer quality bread [39,40].

The mountain rye flours (Table 2) were characterized by low activity of amylolytic enzymes, which was illustrated by the relatively high value of the falling number. A comparison of the results allowed us to notice that the effect of the grain moisture content on the value of the flour falling number was statistically significant, because when comparing wholegrain flours (MC) and flours with bran (MO), a lower value of the falling number was noted in the studies of flours from grain with a lower moisture content (S). In this mountain rye grain, the cover was crushed more, which resulted in a better release of enzymes, and, as a result, the falling number of the obtained flours was lower than in the flours from conditioned grain. Słowik [41] showed that a well-selected rye flour for baking should be characterized by an average amylolytic activity corresponding to the falling number in the range of 125–200 s. However, the tested krzyca rye flours (Table 2) did not meet this criterion, but are similar to the results of studies on rye flour by other authors [40].

A common method used to determine the baking value of rye flour is the amylographic evaluation. The beginning gelatinization temperature in the discussed studies of rye flours from the krzyca rye grain (Table 2) ranged from 55.4 to 57.8 °C and was within the range noted in the studies by Gudmunsson et al. [42] of 54.8 to 60.3 °C. In the studies of Warechowska et al. [23], the beginning gelatinization temperature of the flour suspension from the primitive rye grain was noted to be 48.8 °C. In the discussed studies of rye flours from the mountain rye grain (Table 2), statistical differences were shown between flours of the same type in terms of the end gelatinization temperature, where the value ranged from 73.5 to 79.1 °C. This parameter was statistically significantly different among the krzyca rye flours tested. Rye flour intended for baking bread should be characterized by an end gelatinization temperature in the range of 63–67 °C [43], while all the tested rye flours made from rye grain were characterized by an end gelatinization temperature that was higher (Table 2) than the abovementioned range, which may also be related to the species factor and the method of grinding grains with different moisture contents.

The statistical analysis of the results of the maximum viscosity measurement showed significant differences, and only flours made from conditioned grain (K MC and K MO) did not differ from each other (Table 2). The highest values were obtained in the tests of flours S MBO (1118 AU) and K MBO (1263 AU). The tested extract flours (without bran) (Table 2) should have a higher starch content, due to the dependence of the share of grain components that pass into the flour depending on the flour extract [34], which affects the amylographic curves obtained from the tests of suspensions of the above-mentioned flours. According to Jakubczyk and Haber [28], the maximum viscosity of the rye flour suspension exceeding 700 AU contributes to obtaining poorly loosened bread, while the flour has good baking properties and is suitable for the production of sourdough bread and mixed yeast bread when the maximum value of starch paste is from 350 to 650 AU. The remaining tested rye flours from krzyca rye grain (Table 2) showed values of the parameter in question within the given range of values. The obtained results are similar to the tests of flour from primitive rye grain [23].

### 3.4. Baking Process and Quality of the Tested Mountain Rye Breads

Table 3 presents the results of calculations of the basic parameters of the baking process of the tested mountain rye breads.

Based on the statistical analysis of the obtained research results (Table 3), it was found that the yield of dough intended for baking bread varied, with wholegrain rye flour and flour with bran showing a higher dough yield, both in direct baking with and without dough acidification. In the studies of mountain rye flours, the influence of the dough preparation method on its yield was noted, as wholegrain flours and flours with bran showed a higher yield of dough produced using the method without acidification. In this case, the higher pH of the dough had a lesser effect on the properties of pentosans and proteins than in the matrix of the dough fermented with the addition of lactic acid. Conversely, a higher yield of dough made from krzyca rye flours without bran was obtained using the method where dough was fermented with acidification. The noted observations can be explained by the different chemical compositions of the flours and the degree of granulation, including the degree of starch damage [33,34,38], as wholegrain flours and flours with bran also contained those parts of the grain that are rich in ingredients with high water absorption, such as fiber and especially pentosans [13,14].

Oven loss differences were observed when baking mountain rye breads made from dough fermented with lactic acid addition and without acidification (Table 3). The value of the discussed parameter ranged from 9.4 to 14.4%. S MBO bread made from dough with lactic acid acidification and S MC bread (without acidification) were characterized by the highest value of the tested parameter (14.4%) and were statistically different from the bread made from dough without lactic acid addition obtained from flour without bran from grain conditioned with K MBO (9.4%). However, no statistical differences were observed between the other tested krzyca rye breads.

A comparison of the results for bread yield showed that the yield of bread in both cases (S MC and K MC) with and without lactic acid acidification differed statistically significantly. The value of this parameter ranged from 133.8 to 142.8% and was higher in the case of using wholemeal flour or flour with bran. Significant differences were observed in this parameter between S MO breads (from dough with lactic acid acidification) and S MBO breads (from dough without lactic acid acidification). Similarly to the case of dough yield, the higher yield of bread from wholemeal flour and flour with bran can also be justified by the higher contents of flour components with high water absorption properties [15,16].

Wholegrain breads obtained both with and without lactic acid acidification achieved the best results in terms of the tested baking process parameters. Comparing both variants of the single-phase method, the method of making dough without acidification turned out to be better, because the values of the tested parameters were less diverse.

**Figure 4:**
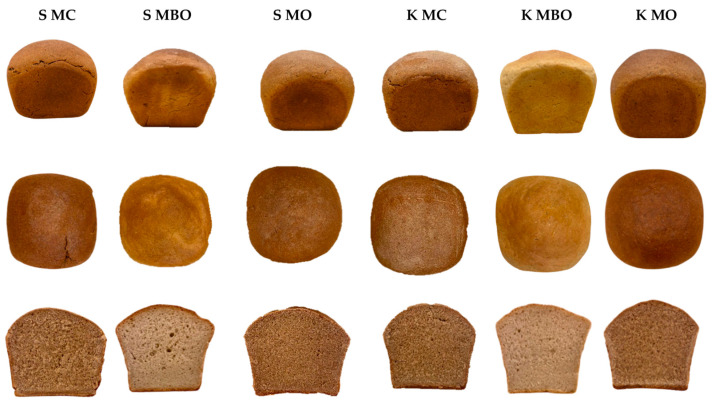
The appearance of the loaves and the crumb of mountain rye bread made with the lactic acid acidification method (A). Abbreviations: S MC—bread made of wholegrain flour from grinding unconditioned grain, S MBO—bread made of extract flour from milling unconditioned grain, S MO—bread made of flour made from a combination of extract flour and bran after milling unconditioned grain, K MC—bread made of wholegrain flour from grinding conditioned grain, K MBO—bread made of extract flour from milling conditioned grain, K MO—bread made of flour made from a combination of extract flour and bran after milling conditioned grain.

**Figure 5:**
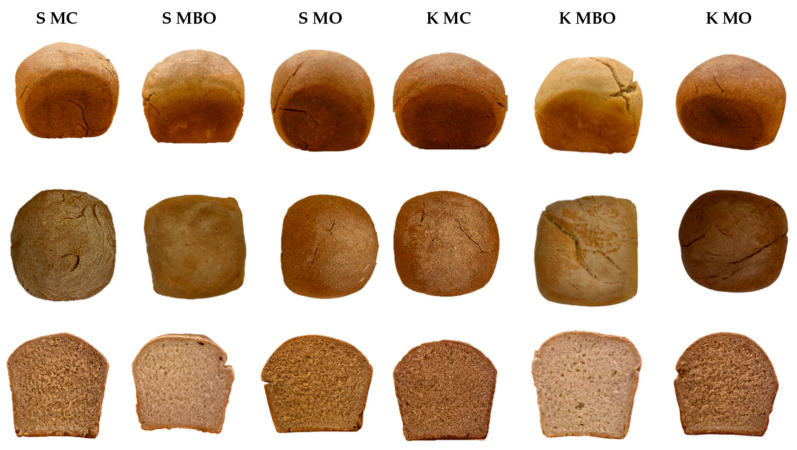
The appearance of the loaves and the crumb of mountain rye bread made with the method without lactic acid acidification (NA). Abbreviations: S MC—bread made of wholegrain flour from grinding unconditioned grain, S MBO—bread made of extract flour from milling unconditioned grain, S MO—bread made of flour made from a combination of extract flour and bran after milling unconditioned grain, K MC—bread made of wholegrain flour from grinding conditioned grain, K MBO—bread made of extract flour from milling conditioned grain, K MO—bread made of flour made from a combination of extract flour and bran after milling conditioned grain.

The obtained mountain rye breads made from dough fermented with and without lactic acid addition were subjected to a volume determination and analysis of crumb quality parameters such as the moisture content, acidity, and porosity. The results of the tests are presented in Table 4. The appearance of the loaves and crumb of the all tested mountain rye breads is presented in Figure 4 and Figure 5.

The moisture content of the crumb of the tested rye breads made from doughs with and without lactic acid acidification was similar. Only the S MC rye bread had the smallest moisture content (45%) and was the only one to differ statistically significantly from the other tested breads (Table 4). Similar moisture content values were obtained by Stępniewska [35] in studies evaluating the baking value of type 500 rye flour.

The acidity of the tested rye bread ranged from 1.8 to 7.5° acidity. Breads made from dough without acidification with lactic acid, K MBO (1.8° acidity) and S MBO (2.6° acidity), were characterized by the lowest acidity. The bread made from dough acidified with lactic acid (S MC) was characterized by the highest acidity (7.5° acidity). The abovementioned breads and the K MBO bread with acidification were statistically different in terms of the acidity of the crumb from all other krzyca rye breads tested. Świderski [44] indicates that the acidity of good quality rye bread should be at a level of 8–11°. The tested rye breads (Table 4) were characterized by lower acidity than in the mentioned literature sources [44], which could be due to the limited possibility of acidifying the dough in the single-phase fermentation method.

In the evaluation of the crumb porosity of the rye breads tested, only the S MC bread (42%) acidified with lactic acid and the S MBO bread (59%) without acidification with lactic acid differed significantly in terms of the parameter discussed. In the case of this parameter, acidification did not significantly affect the porosity of the mountain rye bread, which can be seen in Figure 4 and Figure 5. The value of this parameter in the crumb tests of the other tested rye breads ranged from 43 to 55%. The porosity of the bread depends on the type of bread and is 55÷70% for rye bread and indicates the course of dough fermentation and the baking properties of the flour [45]. In the tests of rye breads made from rye flour from krzyca rye (Table 4), it was shown that the porosity was below the recommended values, which may also indicate that the quality of the finished product is also shaped by the selection of technological parameters. It can be assumed that multiphase fermentation could result in a product with better porosity.

The next parameter analyzed was the specific volume of the tested mountain rye bread, which ranged from 1.35 to 1.93 cm^3^ (Table 4). The statistical analysis revealed significant differences in the values of this parameter between S MBO and K MBO breads (acidified with lactic acid), with S MBO (without acidification) showing the highest values, and S MO (acidified with lactic acid) and K MO (without acidification) breads achieving the lowest values of this parameter. The content of grain cover particles in the flour and the preparation of grain for milling could be the reasons for the differences in the specific volume of the krzyca rye breads, as they affect the chemical composition of the flour and the degree of starch damage. The acidification used in the dough fermentation method contributed insignificantly to the differences in the discussed parameter between the rye breads tested. Similarly, in the studies of other authors [46], larger volumes of wheat bread made of wholegrain flour were not obtained using sourdough compared to baker’s yeast, and the importance of optimizing bread dough recipes and bread baking processes was emphasized.

### 3.5. Organoleptic Evaluation and Quality Classification of the Tested Rye Breads

**Figure 6:**
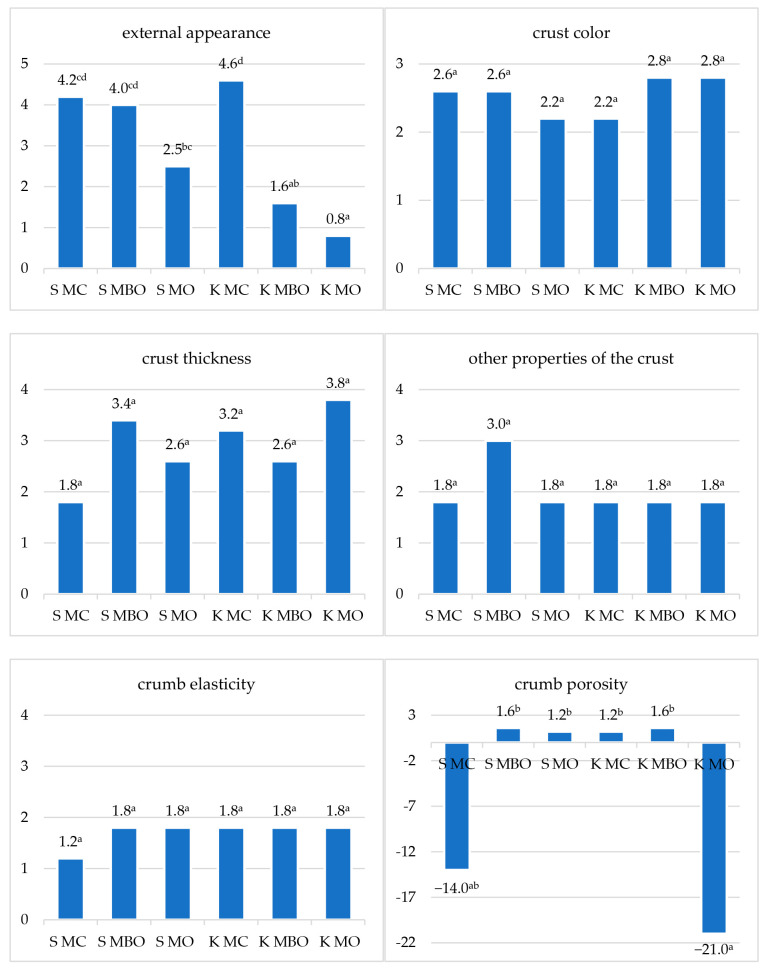

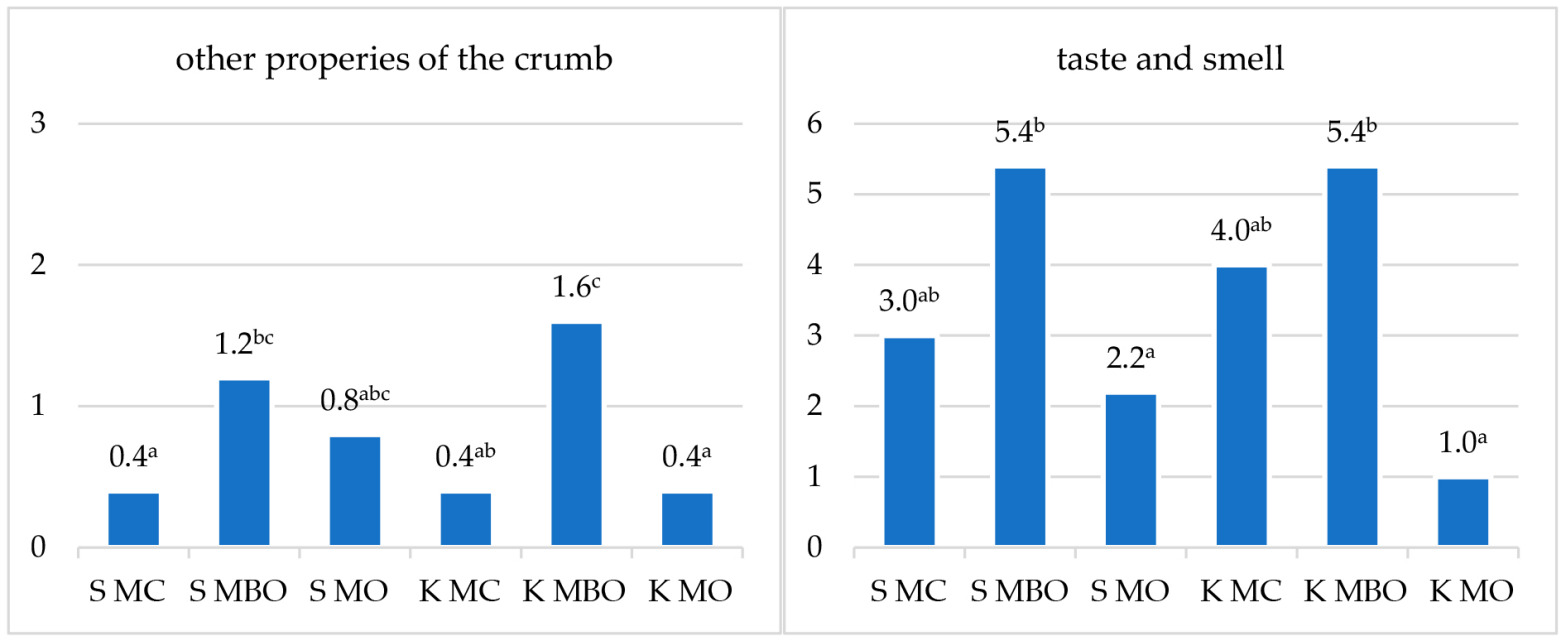
Results of the organoleptic evaluation of rye bread with lactic acid acidification (A). Mean values in columns marked with different letters differ statistically significantly at a significance level of *p* ≤ 0.05. Abbreviations: S MC—bread made of whole grain flour from milling unconditioned grain, S MBO—bread made of extract flour from milling unconditioned grain, S MO—bread made of flour made from a combination of extract flour and bran after milling unconditioned grain, K MC—bread made of whole grain flour from milling conditioned grain, K MBO—bread made of extract flour from milling conditioned grain, K MO—bread made of flour made from a combination of extract flour and bran after milling conditioned grain.

**Figure 7:**
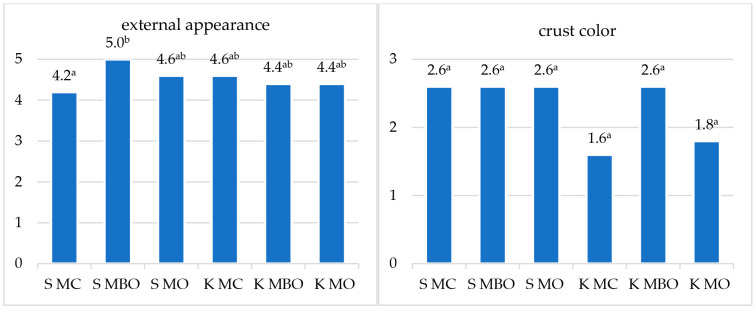

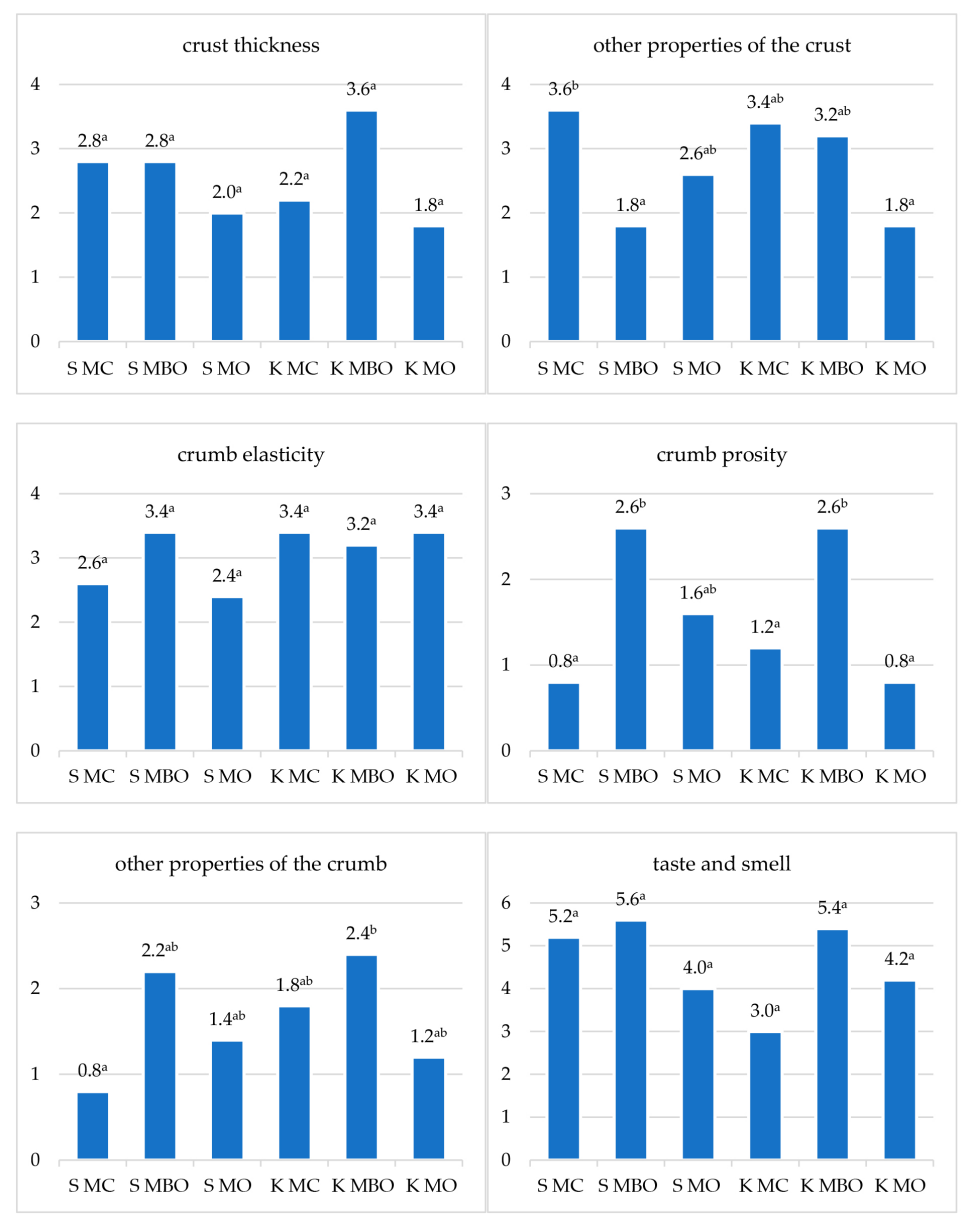
Results of the organoleptic evaluation of rye bread without lactic acid acidification (NA). Mean values in columns marked with different letters differ statistically significantly at a significance level of *p* ≤ 0.05. Abbreviations: S MC—bread made of wholegrain flour from grinding unconditioned grain, S MBO—bread made of extract flour from milling unconditioned grain, S MO—bread made of flour made from a combination of extract flour and bran after milling unconditioned grain, K MC—bread made of wholegrain flour from grinding conditioned grain, K MBO—bread made of extract flour from milling conditioned grain, K MO—bread made of flour made from a combination of extract flour and bran after milling conditioned grain.

The results of the organoleptic evaluation of the tested rye breads obtained from dough fermented with lactic acid and without acidification are presented in the Figure 6 and Figure 7. The summary of the scoring assessment and quality classification of the tested rye breads is presented in Table 5.

The statistical analysis of the results of the evaluation of sensory characteristics of the tested breads made from dough with lactic acid-fermented sourdough confirmed the differences in the external appearance, porosity of the crumb, and other crumb characteristics, as well as the taste and smell, under the influence of the type of rye flour used. The breads made from extract flour were assessed more favorably.

In the case of breads made from dough without additional lactic acid acidification, significant differences were confirmed in the external appearance of the bread, other crust features, porosity of the crumb, and other crumb features. Similar to the method with dough acidification with lactic acid, among the breads obtained in the second variant of dough preparation, the bread made from extract flour was assessed as the best.

The quality classification of breads obtained from dough made using the lactic acid method varied. Two of the tested breads, S MC and K MC, were disqualified due to points below 8 (Table 5). The S MO bread received quality level IV, while the K MC bread received slightly more points, which allowed it to be classified as a bread with level III quality. Breads obtained from extract flour from unconditioned and conditioned grain received the most points and obtained quality level II. None of the tested breads were classified as quality level I. The quality of rye breads without acidification was classified better, only the S MBO and K MBO breads obtained quality level II, while the remaining breads were classified as quality level III. Breads from extract flour (S MBO and K MBO), which obtained quality level II, can be considered the best among breads without lactic acid acidification. Similarly to the breads with acidification, in this case also none of the breads was classified as level I quality.

The results of the sensory evaluation indicate the need to improve the technological process of making bread from rye flour.

### 3.6. Consumer Evaluation of the Tested Rye Breads

**Figure 8:**
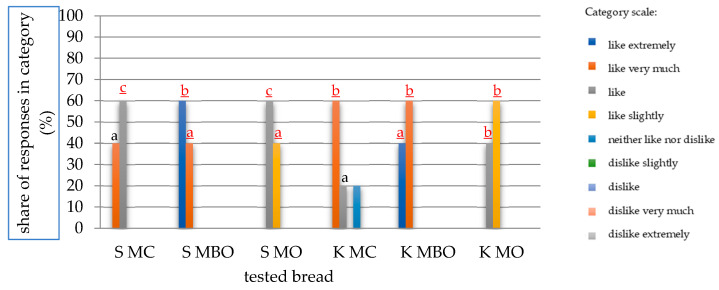
Results of the consumer evaluation of mountain rye bread made with the lactic acid acidification fermentation method (A). The mean values in a given scale category marked with different letters differ statistically significantly at a significance level of *p* ≤ 0.05. Abbreviations: S MC—bread made of wholegrain flour from grinding unconditioned grain, S MBO—bread made of extract flour from milling unconditioned grain, S MO—bread made of flour made from a combination of extract flour and bran after milling unconditioned grain, K MC—bread made of wholegrain flour from grinding conditioned grain, K MBO—bread made of extract flour from milling conditioned grain, K MO—bread made of flour made from a combination of extract flour and bran after milling conditioned grain.

**Figure 9:**
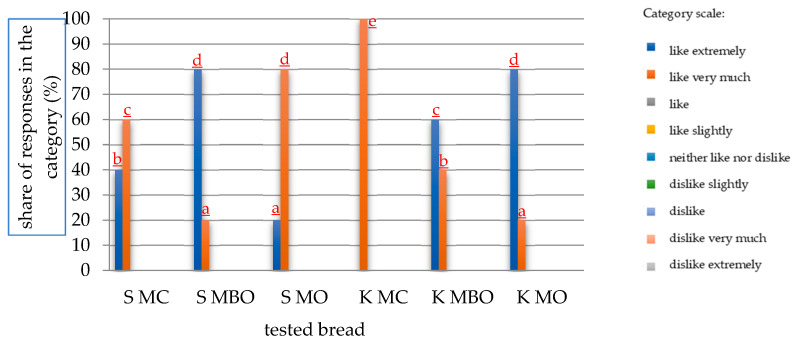
Results of the consumer evaluation of mountain rye bread made with the fermentation method without lactic acid acidification (NA). The mean values in a given scale category marked with different letters differ statistically significantly at a significance level of *p* ≤ 0.05. Abbreviations: S MC—bread made of wholegrain flour from milling unconditioned grain, S MBO—bread made of extract flour from milling unconditioned grain, S MO—bread made of flour made from a combination of extract flour and bran after milling unconditioned grain, K MC—bread made of wholegrain flour from grinding conditioned grain, K MBO—bread made of extract flour from milling conditioned grain, K MO—bread made of flour made from a combination of extract flour and bran after milling conditioned grain.

The tested rye breads were also subjected to a consumer evaluation, and the results are presented in Figure 8 and Figure 9.

According to the panelists’ assessment, the most desirable breads were those made from K MBO and S MBO (without bran) flours, which were the only ones to receive ratings in the “like extremely” category, 40% and 60%, respectively. The remaining ratings were in the “like very much” category. The lowest ratings were given to S MO and K MO breads, whose rating results were divided into the categories “like” and “like slightly”. The ratings for K MC bread turned out to be the most diverse of all, because this bread was the only one to receive responses in three different categories (the others received responses in two categories).

In consumer studies of breads obtained from dough made without lactic acid fermentation, assessments of breads were recorded only in two categories, as the highest percentage of “like extremely” assessments were received by S MBO and K MO breads, which was equal to 80%. The remaining 20% fell in the “like very much” category. K MC bread was the only one to receive 100% of responses in the “like” category. This meant that none of the panelists voted “like extremely” in its case, which is why this bread can be considered the least desirable.

A comparison of the consumer evaluation results of all tested mountain rye breads allowed us to notice that the best consumer desirability was distinguished by breads made of flour without bran obtained by the method of dough fermentation without acidification—S MBO and K MO. The worst evaluation was given to bread made of wholegrain flour from conditioned grain and dough acidification with lactic acid (K MC). Acidification with lactic acid in dough fermentation for rye bread made from mountain rye grain resulted in a worse overall consumer evaluation of the finished product in comparison with breads made from dough without additional acidification with lactic acid.

### 3.7. Graphic Interpretation of the Relationship between the Quality of Bread and the Quality of Rye Flour

**Figure 10:**
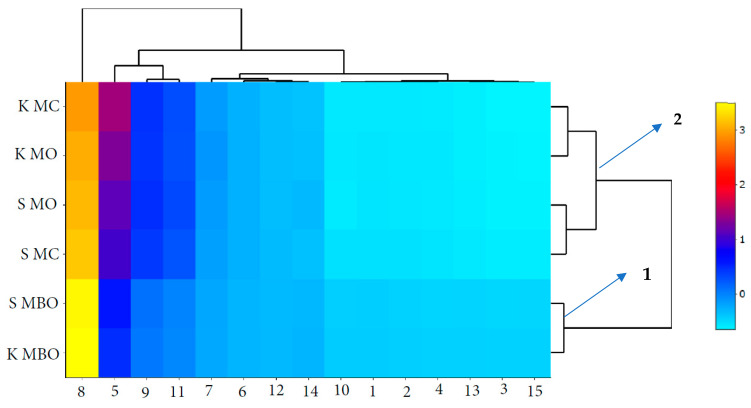
Heat map of the parameters of rye flour and bread made using the method of dough fermentation with lactic acid acidification (A): 1—moisture content in flour (%), 2—crude protein content in flour (% d.m.), 3—total ash content in flour (% d.m.), 4—flour acidity (acidity degree), 5—falling number (s), 6—beginning gelatinization temperature (°C), 7—end gelatinization temperature (°C), 8—maximum viscosity (BU), 9—dough yield (%), 10—oven loss (%), 11—bread yield (%), 12—bread crumb moisture content (%), 13—acidity of bread crumb (acidity degree), 14—porosity of bread crumb (%), and 15—bread specific volume (cm^3^/g). Abbreviations: S MC—bread made of wholegrain flour from grinding unconditioned grain, S MBO—bread made of extract flour from milling unconditioned grain, S MO—bread made of flour made from a combination of extract flour and bran after milling unconditioned grain, K MC—bread made of wholegrain flour from grinding conditioned grain, K MBO—bread made of extract flour from milling conditioned grain, K MO—bread made of flour made from a combination of extract flour and bran after milling conditioned grain.

**Figure 11:**
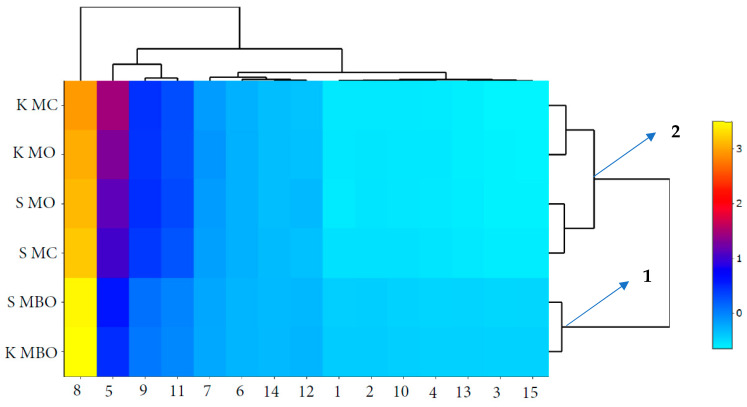
Heat map of the parameters of rye flour and bread made using the method of dough fermentation without lactic acid acidification (NA): 1—moisture content in flour (%), 2—crude protein content in flour (% d.m.), 3—total ash content in flour (% d.m.), 4—flour acidity (acidity degree), 5—falling number (s), 6—beginning gelatinization temperature (°C), 7—end gelatinization temperature (°C), 8—maximum viscosity (BU), 9—dough yield (%), 10—oven loss (%), 11—bread yield (%), 12—bread crumb moisture content (%), 13—acidity of bread crumb (acidity degree), 14—porosity of bread crumb (%), and 15—bread specific volume (cm^3^/g). Abbreviations: S MC—bread made of wholegrain flour from grinding unconditioned grain, S MBO—bread made of extract flour from milling unconditioned grain, S MO—bread made of flour made from a combination of extract flour and bran after milling unconditioned grain, K MC—bread made of wholegrain flour from grinding conditioned grain, K MBO—bread made of extract flour from milling conditioned grain, K MO—bread made of flour made from a combination of extract flour and bran after milling conditioned grain.

As shown in Figure 10 and Figure 11, the parameters tested were grouped using scaled heat maps together with a clustering analysis. The quality of mountain rye baked breads clearly depended on the type of flour used. In particular, rye breads made from flours with bran, both K MBO and S MBO, differed qualitatively from the tested rye breads obtained from the other tested rye flours from krzyca rye grain, as indicated by the grouping of cluster 1. Some differences in the quality parameters of the tested breads, depending on the method of dough fermentation, concerned the parameters oven loss (10) and crumb porosity (14).

## 4. Conclusions

Rye grain (*Secale montanum* Guss.) can be a raw material for the production of flour with good baking value, especially the expected amylographic properties. It was confirmed that the baking value of rye flour obtained from rye grain depends on the preparation of the grain for milling, the method of milling, and the extract of the obtained flour. Rye flours with bran were distinguished by better quality parameters of tested flour, both from grain subjected to conditioning before milling and grain not subjected to this treatment before milling. It was confirmed that the quality of rye bread from mountain rye grain flour depends on the baking value of the flour—including the flour extract—and the methods of dough fermentation and baking used. All types of rye flour tested allowed a finished product of good quality to be obtained by the direct method of dough fermentation and baking.

In terms of the sensory evaluation and overall acceptability of rye bread, the best were breads made of flour without bran and obtained by the direct method of dough preparation, without dough acidification with lactic acid.

At the same time, the demonstrated differences in the quality of the tested flour types indicate the need to continue research in order to optimize the method of dough fermentation and bread baking, with an emphasis on the verification of multiphase dough preparation methods that are traditional and typical for rye doughs.

## Figures and Tables

**Table 1:** Physicochemical properties of the tested mountain rye flours.

Tested Flour	Moisture Content (%)	Crude Protein Content (% d.m.)	Total Ash Content (% d.m.)	Acidity (Acidity)
S MC	12.55 ^ab^ ± 0.15	11.70 ^b^ ± 0.1	1.65 ^c^ ± 0.01	9.3 ^b^ ± 0.2
S MBO	12.45 ^ab^ ± 0.15	8.70 ^a^ ± 0.0	0.58 ^b^ ± 0.02	4.1 ^a^ ± 0.1
S MO	13.25 ^b^ ± 0.25	11.83 ^b^ ± 0.0	1.72 ^d^ ± 0.03	10.2 ^c^ ± 0.1
K MC	12.05 ^a^ ± 0.55	11.90 ^b^ ± 0.0	1.65 ^c^ ± 0.00	10.8 ^cd^ ± 0.1
K MBO	13.50 ^b^ ± 0.10	8.50 ^a^ ± 0.2	0.49 ^a^ ± 0.02	3.5 ^a^ ± 0.1
K MO	12.95 ^ab^ ± 0.35	11.90 ^b^ ± 0.1	1.70 ^d^ ± 0.01	11.2 ^d^ ± 0.1

Mean values in columns marked with different letters differ statistically significantly at a significance level of *p* ≤ 0.05. Abbreviations: d.m.—dry matter, S MC—whole grain flour from grinding unconditioned grain, S MBO—extract flour from milling unconditioned grain, S MO—flour made from a combination of extract flour and bran after milling unconditioned grain, K MC—wholegrain flour from grinding conditioned grain, K MBO—extract flour from milling conditioned grain, K MO—flour made from a combination of extract flour and bran after milling conditioned grain.

**Table 2:** The results of the amylographic analysis of the tested mountain rye flours.

Tested Flour	Falling Number (s)	Beginning Gelatinization Temperature (°C)	End Gelatinization Temperature (°C)	Maximum Viscosity (BU)
S MC	269 ^a^ ± 4	57.6 ^a^ ± 0.2	73.5 ^a^ ± 0.1	616 ^c^ ± 6
S MBO	304 ^cd^ ± 4	55.4 ^c^ ± 0.3	78.2 ^e^ ± 0.2	1118 ^d^ ± 9
S MO	264 ^a^ ± 7	57.0 ^b^ ± 0.2	74.4 ^b^ ± 0.1	557 ^b^ ± 6
K MC	311 ^d^ ± 5	57.8 ^c^ ± 0.2	75.6 ^c^ ± 0.1	529 ^a^ ± 6
K MBO	298 ^c^ ± 1	55.4 ^a^ ± 0.2	77.1 ^d^ ± 0.1	1263 ^e^ ± 7
K MO	286 ^b^ ± 6	57.1 ^b^ ± 0.2	79.1 ^f^ ± 0.1	539 ^a^ ± 5

Mean values in columns marked with different letters differ statistically significantly at a significance level of *p* ≤ 0.05. Abbreviations: S MC—wholegrain flour from grinding unconditioned grain, S MBO—extract flour from milling unconditioned grain, S MO—flour made from a combination of extract flour and bran after milling unconditioned grain, K MC—wholegrain flour from grinding conditioned grain, K MBO—extract flour from milling conditioned grain, K MO—flour made from a combination of extract flour and bran after milling conditioned grain.

**Table 3:** Baking process parameters of the tested mountain rye breads.

Tested Bread	Dough Yield (%)	Oven Loss (%)	Bread Yield (%)
breads with lactic acid acidification (A)			
S MC	166.1 ^cde^ ± 1.8	12.3 ^abc^ ± 1.6	141.3 ^c^ ± 1.9
S MBO	165.3 ^cd^ ± 0.9	14.4 ^c^ ± 3.9	135.2 ^ab^ ± 1.2
S MO	165.5 ^d^ ± 0.2	9.7 ^ab^ ± 1.3	142.8 ^d^ ± 1.5
K MC	165.2 ^cd^ ± 1.5	11.9 ^abc^ ± 0.9	140.7 ^c^ ± 0.9
K MBO	164.3 ^c^ ± 0.4	12.1 ^abc^ ± 0.2	135.7 ^ab^ ± 1.9
K MO	159.4 ^a^ ± 1.0	11.7 ^abc^ ± 1.0	136.4 ^ab^ ± 0.9
breads without lactic acid acidification (NA)			
S MC	165.4 ^cde^ ± 1.2	14.4 ^c^ ± 0.1	136.2 ^b^ ± 0.2
S MBO	164.2 ^c^ ± 0.3	13.8 ^bc^ ± 0.5	133.8 ^a^ ± 0.2
S MO	165.5 ^cd^ ± 1.4	9.9 ^ab^ ± 0.5	142.4 ^d^ ± 0.2
K MC	166.3 ^e^ ± 0.3	10.4 ^abc^ ± 0.9	141.6 ^cd^ ± 0.9
K MBO	163.2 ^b^ ± 0.3	9.4 ^a^ ± 1.0	139.6 ^c^ ± 1.2
K MO	165.5 ^cd^ ± 1.1	10.8 ^abc^ ± 0.4	141.4 ^c^ ± 0.5

Mean values in columns marked with different letters differ statistically significantly at a significance level of *p* ≤ 0.05. Abbreviations: S MC—bread made of wholegrain flour from grinding unconditioned grain, S MBO—bread made of extract flour from milling unconditioned grain, S MO—bread made of flour made from a combination of extract flour and bran after milling unconditioned grain, K MC—bread made of wholegrain flour from grinding conditioned grain, K MBO—bread made of extract flour from milling conditioned grain, K MO—bread made of flour made from a combination of extract flour and bran after milling conditioned grain.

**Table 4:** Quality parameters of the tested mountain rye breads.

Tested Bread	Moisture (%)	Acidity (Acidity Degree)	Porosity (%)	Specific Volume (cm^3^/1 g)
breads with lactic acid acidification (A)				
S MC	46.5 ^b^ ± 0.1	5.5 ^c^ ± 0.2	42 ^a^ ± 2	1.51 ^ab^ ± 0.10
S MBO	46.1 ^b^ ± 0.1	5.2 ^c^ ± 0.3	50 ^ab^ ± 0	1.93 ^d^ ± 0.05
S MO	46.7 ^b^ ± 0.1	7.5 ^e^ ± 0.2	50 ^ab^ ± 7	1.42 ^a^ ± 0.04
K MC	47.0 ^b^ ± 0.4	6.7 ^de^ ± 0.1	43 ^ab^ ± 6	1.46 ^ab^ ± 0.03
K MBO	47.0 ^b^ ± 0.4	4.1 ^b^ ± 0.0	55 ^ab^ ± 9	168 ^bc^ ± 0.03
K MO	46.1 ^b^ ± 0.1	6.0 ^cd^ ± 0.1	43 ^ab^ ± 1	1.46 ^ab^ ± 0.03
breads without lactic acid acidification (NA)				
S MC	45.0 ^a^ ± 0.4	5.8 ^cd^ ± 0.1	45 ^ab^ ± 2	1.51 ^ab^ ± 0.12
S MBO	46.6 ^b^ ± 0.3	2.6 ^a^ ± 0.1	59 ^b^ ± 1	178 ^cd^ ± 0.11
S MO	46.4 ^b^ ± 0.2	5.8 ^cd^ ± 0.1	48 ^ab^ ± 8	1.38 ^a^ ± 0.08
K MC	46.4 ^b^ ± 0.8	6.1 ^cd^ ± 0.0	48 ^ab^ ± 3	1.50 ^ab^ ± 0.03
K MBO	46.7 ^b^ ± 0.3	1.8 ^a^ ± 0.1	48 ^ab^ ± 4	1.55 ^ab^ ± 0.12
K MO	46.4 ^b^ ± 0.2	6.0 ^cd^ ± 0.0	46 ^ab^ ± 3	1.35 ^a^ ± 0.03

Mean values in columns marked with different letters differ statistically significantly at a significance level of *p* ≤ 0.05. Abbreviations: S MC—bread made of wholegrain flour from grinding unconditioned grain, S MBO—bread made of extract flour from milling unconditioned grain, S MO—bread made of flour made from a combination of extract flour and bran after milling unconditioned grain, K MC—bread made of wholegrain flour from grinding conditioned grain, K MBO—bread made of extract flour from milling conditioned grain, K MO—bread made of flour made from a combination of extract flour and bran after milling conditioned grain.

**Table 5:** Scoring assessment results and quality classification of the tested rye breads.

Tested Bread	Points for Organoleptic Characteristics	Points for Physico-Chemical Characteristics	Total Points	Quality Class of Bread
breads with lactic acid acidification (A)				
S MC	1.0 ^b^ ± 5.4	4	5.0	disqualified
S MBO	24.2 ^e^ ± 1.3	8	32.2	II
S MO	16.7 ^c^ ± 0.6	6	22.7	IV
K MC	20.8 ^d^ ± 1.3	7	27.8	III
K MBO	23.0 ^e^ ± 1.2	8	31.0	II
K MO	−8.4 ^a^ ± 7.6	1	−7.4	disqualified
breads without lactic acid acidification (NA)				
S MC	22.6 ^de^ ± 1.4	8	30.6	III
S MBO	26.0 ^f^ ± 1.3	8	34.0	II
S MO	21.2 ^d^ ± 1.0	8	29.2	III
K MC	21.2 ^d^ ± 1.1	8	29.2	III
K MBO	27.4 ^fg^ ± 1.0	8	35.4	II
K MO	19.4 ^c^ ± 1.3	8	27.4	III

The mean values in a given scale category marked with different letters differ statistically significantly at a significance level of *p* ≤ 0.05. Abbreviations: S MC—bread made of wholegrain flour from grinding unconditioned grain, S MBO—bread made of extract flour from milling unconditioned grain, S MO—bread made of flour made from a combination of extract flour and bran after milling unconditioned grain, K MC—bread made of wholegrain flour from grinding conditioned grain, K MBO—bread made of extract flour from milling conditioned grain, K MO—bread made of flour made from a combination of extract flour and bran after milling conditioned grain.

## Data Availability

The original contributions presented in the study are included in the article, further inquiries can be directed to the corresponding author.
